# Does Venue of HIV Testing and Results Disclosure in the Context of a Research Study Affect Adolescent Health and Behavior? Results from a Study in Western Kenya

**DOI:** 10.3390/ijerph19063249

**Published:** 2022-03-10

**Authors:** Winnie Kavulani Luseno, Samuel H. Field, Bonita J. Iritani, Fredrick S. Odongo, Daniel Kwaro, Stuart Rennie

**Affiliations:** 1 Chapel Hill Center, Pacific Institute for Research and Evaluation (PIRE), Chapel Hill, NC 27514, USA; samfield31@gmail.com (S.H.F.); iritani@pire.org (B.J.I.); 2 Centre for Global Health Research, Kenya Medical Research Institute (KEMRI), Kisumu 1578-40100, Kenya; fodongo1@gmail.com (F.S.O.); kdan2008@gmail.com (D.K.); 3 Department of Social Medicine, University of North Carolina at Chapel Hill, Chapel Hill, NC 27599, USA; stuart_rennie@med.unc.edu

**Keywords:** adolescent research participation, HIV testing and results disclosure, quality of life, depressive symptoms, sexual risk behavior, sub-Saharan Africa, HIV prevention

## Abstract

Ethical concerns about risks to minor adolescents participating in HIV prevention research is a barrier to their inclusion. One concern is whether HIV testing and results disclosure venue affects the health and behavior of adolescent participants. We assessed for differential effects on quality of life (QOL), depressive symptoms, and sexual behavior due to (1) testing venue (home or health facility) and (2) test result (HIV-positive, HIV-negative, indeterminate). We collected data at three timepoints (baseline, 2-month follow-up, 12-month follow-up) from 113 Kenyan adolescents aged 15–19 (51% female). We analyzed the data using linear mixed effects models for the QOL and depressive symptoms outcomes and a logistic model for the sexual behavior outcome. Results showed a small mental health benefit for adolescents tested for HIV at a health facility compared with home. There was little evidence that testing venue influenced sexual behavior or that test results moderated the effects of HIV testing across all outcomes. The decision to conduct HIV testing at home or a health facility may not be very consequential for adolescents’ health and behavior. Findings underscore the need to critically examine assumptions about adolescent vulnerability to better promote responsible conduct of HIV prevention research with youth in sub-Saharan Africa.

## 1. Introduction

There continues to be a pressing need to develop, test, and implement effective HIV prevention and treatment strategies for adolescents in high burden countries [1,2,3,4,5], especially in sub-Saharan Africa where the majority of the world’s adolescents (ages 10–19) living with HIV reside [6,7]. Since the mid-2000s, there have been calls for more HIV-related research among adolescents to fulfill this need [2,5,8,9,10]. However, despite the high public health value of involving adolescents in such research, investigators are sometimes reluctant to include minors under 18 years of age in HIV studies because of ethical concerns about the risks of their participation coupled with the lack of consistent, unambiguous ethical principles to guide HIV-related research among adolescent minors [9,11,12,13,14,15,16].

One important ethical issue pertains to the effects of venue of HIV testing and disclosure of results in the context of HIV prevention research on adolescent health and behavior [17]. Given the known limitations of self-reported sexual behavior, HIV biological measures obtained via testing are important for evaluating the effectiveness of prevention interventions for adolescents. Prior to 2010, the pervading assumption among research stakeholders was that disclosure of HIV test results to adolescent participants would negatively impact their wellbeing and health behavior [17]. Thus, investigators were reluctant to include minor adolescents in HIV prevention studies which conducted HIV testing; those that did used a variety of procedures including not providing participants with their test results, although biological samples were collected for testing by study staff, but instead liaising with voluntary counseling and testing (VCT) sites to provide free testing services to study participants [18,19,20].

A review of recent literature indicates that testing and disclosure of results are now standard practice in HIV prevention studies with community-based adolescent participants [21,22,23,24,25]. Most studies provide facility- (e.g., clinics or hospitals) or community-based (e.g., home or mobile units) HIV testing, counseling, and referral services by trained counselors working as part of the research team. Others liaise with local health facilities to provide standard HIV testing services to study participants and share test results with the research team with participants’ permission. 

However, few studies have examined the effect of the location of HIV testing and results disclosure in the context of research on adolescent wellbeing and behavior. Of the limited research that is available, findings are mixed. One study among females aged 15–25 in Malawi found that after disclosure of test results, condom use increased among both participants with seropositive and seronegative results, consistent condom use was more likely among those with seropositive results, and non-barrier contraceptive use decreased among those with seronegative results due likely to an increase in abstinence [26]. An earlier study in Malawi among never-married females aged 13–22 found no overall effect of home-based HIV testing and counseling on HIV infection, Herpes Simplex Virus 2 (HSV-2) infection, and number of sexual partners at 12-month follow-up [27]. However, analyses stratified by test results found an increase in the probability of HSV-2 infection at follow-up among those who tested positive for HIV at baseline. We did not find any studies with adolescent male study participants or examining the effects of HIV testing and disclosure of results on adolescent mental health outcomes. We also did not find any studies examining whether there are differential effects of testing venue (e.g., health facility versus home-based testing) on adolescent health outcomes. 

In this paper, we used longitudinal data from 113 Kenyan female and male adolescents aged 15–19 years to examine the effect of where HIV testing and disclosure of results occurs on the quality of life (QOL), mental health, and sexual behavior of adolescent research participants. Specifically, we examined whether effects differed depending on whether adolescents received HIV testing and test results at home or at a health facility. We also investigated whether effects on wellbeing and behavior differed by adolescents’ test results (HIV-positive, HIV-negative, and indeterminate/inconclusive). Our study findings may inform best practices for testing and disclosure of results among adolescent participants in future HIV prevention research as well as guidelines for the ethical conduct of adolescent HIV research in sub-Saharan Africa. Our findings may also be useful for HIV prevention programmers. 

## 2. Materials and Methods

### 2.1. Design, Participants, and Procedures

Data are from baseline and follow-up data collection sessions of a hybrid design study with experimental (cluster randomization of villages to testing venue) components embedded within an observational cohort study. The overall goal of the study was to examine ethical issues in adolescent HIV-related research. Additional details about study design and procedures were described in earlier publications [28,29]. The study was conducted between June 2016 and December 2018. At baseline, participating adolescents completed a survey and were tested for HIV. Follow-up surveys were conducted 2–4 months post-baseline (referred to in this paper as the “2-month follow-up”) and 12–15 months post-baseline (i.e., the “12-month follow-up”). 

The study was conducted in a rural county in the Nyanza region of western Kenya. HIV prevalence among young people aged 15–24 years in the study county is among the highest in the country, at 7.2% among young women and 4.5% among young men, compared with 2.34% and 1.3%, respectively, at the national level [30]. Three sub-counties comprised strata. A total of 119 villages or village clusters were randomly chosen for recruitment out of a total sample frame of 254 villages/village clusters. Of the 119 villages/village clusters chosen, 90 of them ended up having an enrolled participant. Village clusters of participants were randomly assigned to take the baseline survey and HIV test at either a nearby clinic or the adolescent’s home. Random assignment of clusters occurred during community meetings held at the three sub-counties whereby local community stakeholders drew numbers corresponding to villages out of a basket. Concealment of assignment allocation and blinding of research staff and participants to treatment assignment were not performed.

We used posters and village sensitization meetings in the sampled villages to recruit eligible adolescents. We continued our recruitment effort in the communities until we obtained our target numbers. We sought to enroll equal numbers of participants by gender, sub-county, and age group (15–17 years and 18–19 years). Staff screened adolescents for study eligibility. To be eligible, adolescents had to be between 15–19 years old, not have been tested for HIV in the past six months, and should never have tested positive for HIV. Of 6726 adolescents assessed for eligibility, 1927 were ineligible and 703 either did not participate in the baseline survey or their baseline data were excluded. The final baseline sample was 4096 [28].

Following completion of the baseline survey, a trained counselor conducted HIV testing (using finger prick blood), provided pre-/post-test counseling, and disclosed test results based on national guidelines [31]. The same procedures were used to conduct HIV testing and results disclosure for all participants. Sequential testing was conducted using rapid HIV test kits (Determine (Determine™, Abbott Laboratories, Chicago, IL, USA) for screening and, if positive, First Response (Premier Medical Corporation, Kachigam, India) for confirmatory testing). Adolescents aged 15–17 were required to have a parent or guardian over the age of 18 present with them during baseline procedures (i.e., data collection and HIV testing). Each adolescent participant was given the choice of whether their test result should be disclosed to them while their supporting adult was present or given to their supporting adult separately. Adolescents aged 18–19 were encouraged but not required to have a supportive adult with them. All participating adolescents received their test results (positive, negative, or indeterminate) at baseline. Participants with positive results were referred to a health facility of their choice for treatment and support services. All participants with indeterminate (inconclusive) results were successfully referred to a health facility of their choice for further testing services including laboratory testing. By the end of the study, all participants with indeterminate results at baseline had received confirmatory laboratory testing indicating they were HIV-negative. (We refer to this group as participants with “indeterminate” results because they had received indeterminate baseline results.) The timeframe in which participants with indeterminate baseline results received their confirmatory results varied due to factors beyond our control. These factors included when, after referral, participants went to the clinic to have a blood sample drawn for laboratory testing, when the health facility sent the blood sample to the laboratory for testing, when the laboratory did the confirmatory test and sent the results to health facility staff, and when health facility staff informed the participants. About half of the participants had received their results by the 2-month follow-up session and the rest had received their results by the 12-month follow-up session. A goal of this study is to examine whether, compared with participants who received positive and negative test results, wellbeing and behavior outcomes differed at the 2-month and 12-month follow-up sessions for participants who received indeterminate results at baseline. 

All participants who had positive or indeterminate HIV test results at baseline were selected to participate in the 2-month and 12-month follow-up surveys. A sample of participants who received HIV-negative results (*n* = 70) was systematically selected (every 30th in their demographic category) and stratified to ensure diversity by gender, age, testing venue (home or clinic), and sub-county. They were then recruited for a 2-month follow-up session. Among the HIV-negative adolescents at baseline that participated in the 2-month follow-up, a subset (due to budget and timeline constraints) was randomly selected to be recruited for a 12-month follow-up session (*n* = 31). Selection for this HIV-negative subsample was conducted by stratifying by gender, age group (15–17 years vs. 18–19 years), sub-county, and testing venue. Participants also needed to be able to attend the 12-month follow-up session within the 12–15 months window. Staff maintained contact information to locate and schedule participants for the 2-month and 12-month follow-up sessions. 

At all three timepoints, survey questionnaires were administered individually using an audio computer–assisted self–interview (ACASI) format in the participant’s language of preference (English, Luo, Kiswahili) and at a location that provided privacy and confidentiality (parent/guardian not present). Follow-up data collection occurred at the participant’s home or a nearby location. Separate versions of follow-up survey questionnaires were developed for participants who had received HIV-positive results versus those who had received an HIV-negative or indeterminate result at baseline. All participants received a t-shirt on completion of baseline activities. Those who were assigned to receive HIV testing at the clinic also received KSh300 (~USD3) as reimbursement for travel costs. On completion of the 2-month and 12-month follow-up sessions, participants received KSh400 (~USD4) and KSh500 (~USD5), respectively. 

### 2.2. Human Subjects Protections

Participation in the study was voluntary. Adolescents provided verbal consent prior to answering the screening questionnaire. Before baseline data collection, adolescents aged 18–19 years signed a written informed consent form. For adolescents aged 15–17 years old, we obtained written consent from their parent or guardian as well as child assent. A waiver of parent/guardian consent was used in the case of emancipated minors (e.g., married or cohabiting, pregnant or a parent, or living in a child-headed household). Study staff reviewed consent form information with participants prior to each follow-up session.

### 2.3. Measures

Data used in the analyses were from the eligibility screening questionnaire, baseline survey, baseline HIV testing results, 2-month follow-up survey, and 12-month follow-up survey. The primary purpose of our study was to examine the effects of venue of HIV testing and results disclosure coded as clinic or home (the clustered randomized treatment assignment). Our secondary purpose was to examine the effect of the baseline HIV test result coded negative, positive, or indeterminate. Thus, the primary exposure variable was whether the testing and results disclosure venue was clinic or home, and the secondary exposure variable was receiving a negative, positive or indeterminate test result. In the analyses, timepoint refers to the baseline, 2-month follow-up, or 12-month follow-up data collection. Village cluster was the adolescent’s residential location (individual village or a cluster of villages) at baseline.

Outcome measures were created for each of the three timepoints. Depressive symptoms were based on responses to the Center for Epidemiological Studies Depression Scale Revised (CESD-R), an instrument measuring current depression symptomatology and dysphoria [32]. A composite CESD-R score was created by summing the 20 items in the scale (coded from 0–3, lowest to highest symptom frequency) yielding a possible range of 0–60 for the overall score, with higher scores indicating higher levels of depression. Reliability for the scale in our full baseline sample was Cronbach’s alpha = 0.92.

QOL items were from the 26-item World Health Organization Quality of Life Questionnaire abbreviated version (WHOQOL-BREF) [33,34]. Response options were on a 5-point Likert scale that instructed respondents to think about their life in the last four weeks. Procedures used to create and assess the psychometric performance of the QOL measures in our study sample and psychometric results are described in an earlier publication [28]. Two QOL scales (possible range = 1–5, with higher scores indicating better QOL) were created based on factor analysis results: social-physical health (mean of 10 items, baseline Cronbach’s alpha = 0.83) and psychological-environmental QOL (9 items, baseline alpha = 0.76) [28].

HIV sexual risk behavior questions were used to create a composite, ordinal 3-category variable. At baseline, the variable was coded 0 = never had sex (Least risk), 1 = used condom during last sexual intercourse and had fewer than two partners in the past 12 months (Less risky), and 2 = no condom use during last sexual intercourse and/or had two or more partners in past 12 months (More risky). The variables for each follow-up session were coded 0 = has not had sex since baseline or never had sex (Least risk), 1 = used condom during last sexual intercourse and had fewer than two sex partners since baseline (Less risky), and 2 = no condom use during last sexual intercourse and/or had two or more partners since baseline (More risky).

Measures used to describe sample characteristics included gender (male, female), age, ever been pregnant or impregnated a partner (no, yes), orphan status (one or both parents deceased versus neither deceased), currently enrolled in school or completed secondary school (no, yes), and ever been married (no, yes). Religious service attendance of once a week or more frequently was coded yes or no. Religious affiliation was Roman Catholic; Protestant or other Christian; or Muslim, no religion, or other. Sub-county location reflected the participants’ residence (labeled “1,” “2”, and “3”).

### 2.4. Analyses

Data analyses were conducted using SAS (version 9.4; SAS Institute Inc, Cary, NC, USA) and R (version 4.1.0; The R Foundation, Vienna, Austria). From the baseline data, we extracted the age and gender of each subject, cluster (village) membership, clustered treatment assignment (home vs. clinic), and baseline measures of each study outcome. Our analyses described below was based on our primary research question: Is testing site (home vs. clinic) associated with our primary outcomes (depressive symptoms, QOL, HIV sexual risk behavior)? Although the HIV testing site was randomly assigned by village cluster, participants were aware of their assignment prior to completing the baseline survey. We therefore assessed the impact of HIV testing site at all three measurement occasions (baseline, 2-month follow-up, 12-month follow-up).

We used a linear mixed effects model for the QOL and depressive symptoms outcomes. Our assessment was based on the following model: Let yi,j,t equal one of the three outcome measures for subject i in cluster j at time t. Where i ∈ (1, 2, …, *n*) subjects, j ∈ (1, 2, …, *m*) clusters, and t ∈ (0, 2, 12) months.

yi,j,t = θi,j,t + μj + ϵi,j,t

For the mean model, θi,j,t, we employed dummy variable coding for (1) measurement occasion, (2) cluster-level treatment, and (3) HIV test result. The model includes all possible two-way and three-way interactions. The residual component of the model includes a normally distributed, cluster-level random effect, μj ~N(0,τ2), and a within subject residual, ϵi,j,t, where ϵi,j,t is assumed to be drawn from a multivariate normal distribution with an unstructured variance-covariance matrix. This specification allows for heteroskedastic within-subject variance at each measurement occasion and different within-subject correlations between each measurement occasion.

We retained all 113 subjects in the analyses, regardless of how many follow-up sessions they had completed. Indeed, one of the advantages of our model is that it does not require complete case analysis, which can lead to substantially biased treatment effect estimates [35]. In our model, estimates of the fixed effect parameters remain unbiased under the missing at random assumption (MAR) assumption, which states that the likelihood of a missing observation for each subject is independent of its “true” value, conditional on subjects observed data [35].

To evaluate model fit, we created mean plots from the estimated models and overlayed plots of the observed data. We performed type III statistical tests for the model fixed effects. Rather than adopting a strict statistical significance threshold (e.g., *p* < 0.05), we report the *p*-values out to three significant digits and conduct follow up analyses on home vs. clinic comparisons that are suggestive. We also confined our attention to those model effects that include the treatment indicator (clinic vs. home) and report point, and interval estimates as standardized mean differences (SMD), calculated by dividing the estimated difference in means by the square root of the within-subject variance components. When an estimate involved averaging over all three measurement occasions, we pooled/averaged the variance components for each measurement occasion before taking the square root. All effect estimates that averaged over the levels of predictors in the model not represented in the comparison (i.e., measurement occasion and baseline test result) were uniformly weighted, irrespective of the sample sizes for each level. Finally, we employed the conventional Cohen’s D thresholds to characterize the size of the estimated effect as “small” (SMD < 0.2), “medium” (0.2 < SMD < 0.5) or large (SMD > 0.8).

To accommodate the three-level ordinal sexual behavior measure, we applied a cumulative logistic specification. We coded the variable such that positive effects (i.e., odds ratio > 1) indicated less risky sexual behavior. To simplify the specification of the subject-level residual component, we dropped the unstructured residual error structure and included a subject-level random effect instead. This random effect specification means that the parameter estimates for the fixed effects (e.g., mean plots) have a “subject-specific” rather than a “population averaged” interpretation [36]. Finally, the cross-classification of the risk behavior outcome and the other predictors in the model resulted in zero cases in some cells. This caused estimated parameters that represent contrasts between some cell means to approach infinity. We therefore dropped the baseline test result predictor and only included testing site, measurement occasion, and their two-way interaction.

## 3. Results

**Figure 1:**
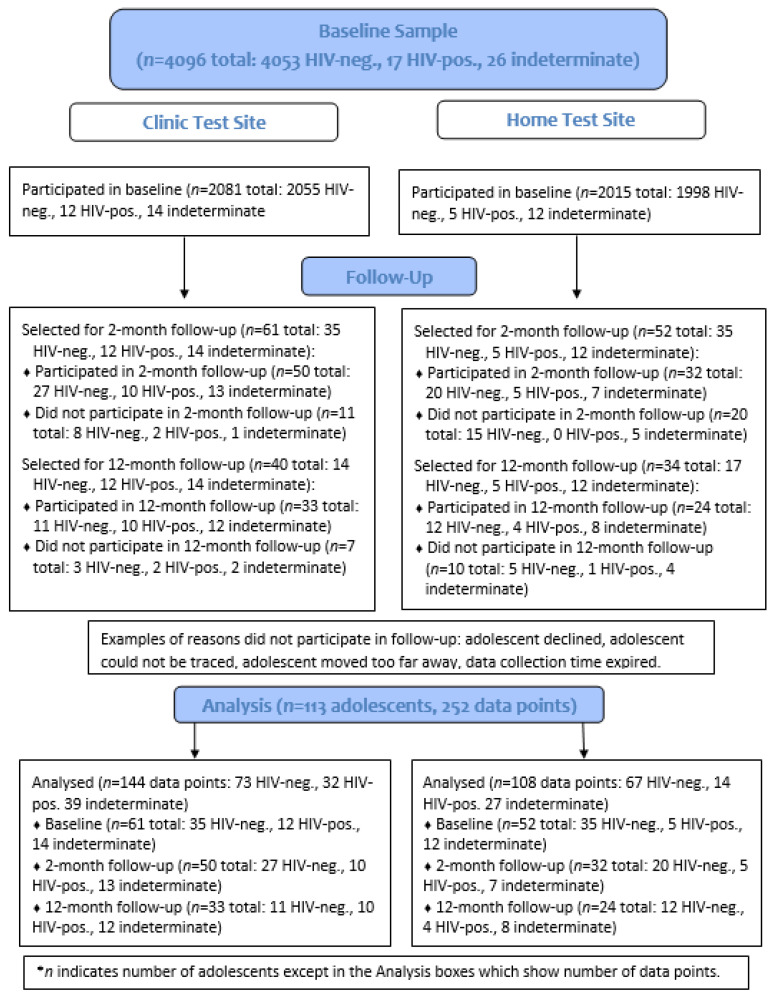
Case Flow Diagram of Study Participants.

Numbers of baseline participants recruited for follow-up (all with positive and indeterminate HIV test results and a 1.7% sample of those with negative results) and resulting numbers with survey data are shown in Figure 1. The analytical sample comprised the 113 adolescents who had been selected for follow-up sessions from the clinic and home testing sites (17 HIV-positive, 26 indeterminate, and 70 HIV-negative at baseline). The numbers of youth that participated in the 2-month follow-up were 15 out of 17 HIV-positive (88.2%), 20 out of 26 indeterminate (76.9%), and 47 out of 70 HIV-negative (67.1%). Participation in the 12-month follow-up was as follows: 14 out of the 17 HIV-positive participants (82.4%), 20 out of 26 indeterminate participants (76.9%), and 23 out of 31 HIV-negative participants recruited (74.2%). Overall, 82 of 113 selected youth (72.6%) and 57 of 74 selected youth (77.0%) participated in the 2-month follow-up and 12-month follow-up, respectively. The 113 participants provided a total of 252 records of data across the timepoints.

Table 1 presents baseline characteristics of the full baseline sample and the sample of respondents who participated in either one or both follow-up sessions. The two groups were similar in all characteristics.

**Figure 2:**
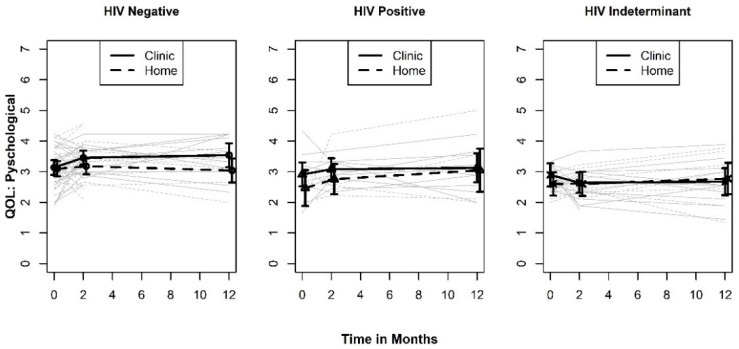
Plots of QOL Psy by HIV test results.

The mean plots for the psychological environment QOL measure shown in Figure 2 suggest a small benefit for testing in the clinic for two out of the three groups (HIV negative and positive test results). However, except for the main effect (*p* = 0.171), none of the type III tests we conducted involving testing site approached conventional levels of statistical significance. The least squares mean point and interval estimates for the main effect of test location is SMD = 0.328 (95% Confidence Interval [CI]: −0.143, 0.8). This is a “medium” effect with 95% CIs that include the null hypothesis but exclude both “large”, positive effects of being tested at a clinic (SMD > 0.8) and medium to large effects in the opposite direction. On the other hand, we cannot rule out the possibility that the “true” effect of testing site is either zero or negligible in magnitude in either direction.

**Figure 3:**
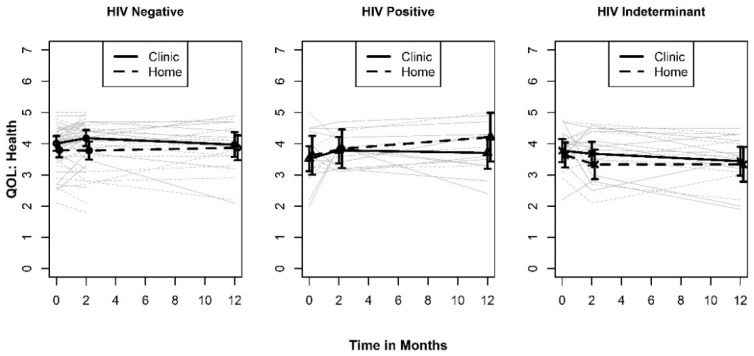
Plots of QOL Health by HIV test result.

**Figure 4:**
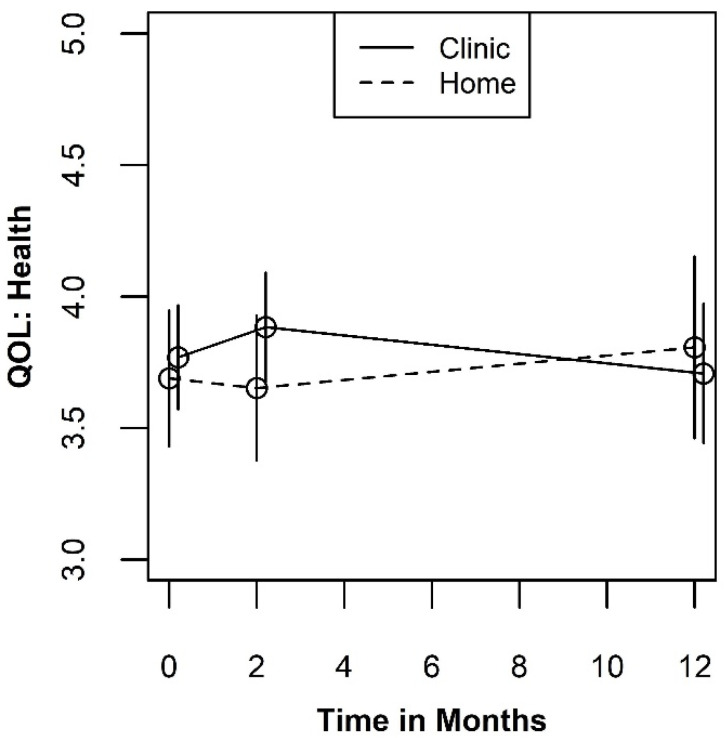
Plot of the two-way interaction for QOL Health by time.

Like the psychological-environmental QOL, the mean plots for the social-physical health QOL measure shown in Figure 3 revealed that the differences between the testing site groups are small relative to the width of the standard errors. However, the type III tests we conducted suggested the presence of a two-way interaction between testing site and measurement occasion (*p* = 0.105). A least squares mean plot of the two-way interaction is shown in Figure 4. When averaged over the three test result categories, the pattern of differences between the levels of testing sites indicates an initial benefit of testing in a clinic at two months (SMD = 0.329; 95% CI: −0.163, 0.821). Although this effect is in the same direction and is similar in magnitude to what was observed in the psychological-environmental QOL, it is not sustained over the 12-month study window. In addition, the confidence intervals for the effect at two months does not exclude the null hypothesis or the possibility that the “true” effect size is small and favors testing at home rather than a clinic.

**Figure 5:**
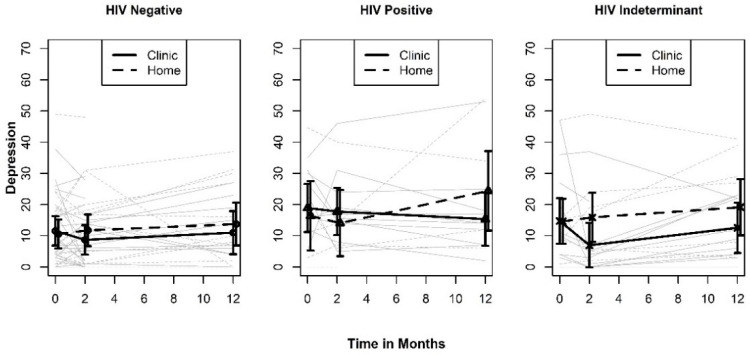
Plots of depression by HIV test result.

**Figure 6:**
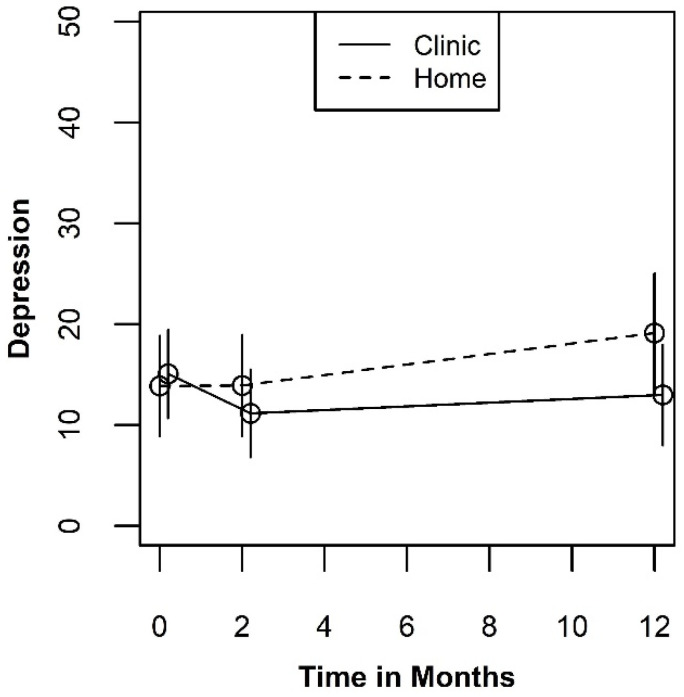
Plot of the two-way interaction for depression by time.

The mean plots for depression shown in Figure 5 indicate a more consistent benefit for testing at the clinic than was observed for either of the two QOL measures. The Type III test results point to a potentially significant two-way interaction between timepoint and testing site (*p* = 0.078). A least squares mean plot of the two-way interaction is shown in Figure 6. When averaged over the three test result categories, the pattern of differences between the levels of testing sites indicates a benefit of testing in a clinic that is largest at the 12-month measurement occasion (SMD = −0.467; 95% CI: −1.057, 0.123). This effect is larger than either of the effects we observed for the QOL outcomes but would still be considered a “medium” effect size. If real, the appearance of a benefit for testing at clinic at 12 months points to a psychological process that unfolds gradually over time. None-the-less, these results should be interpreted with caution since the confidence intervals also include the null effect.

**Figure 7:**
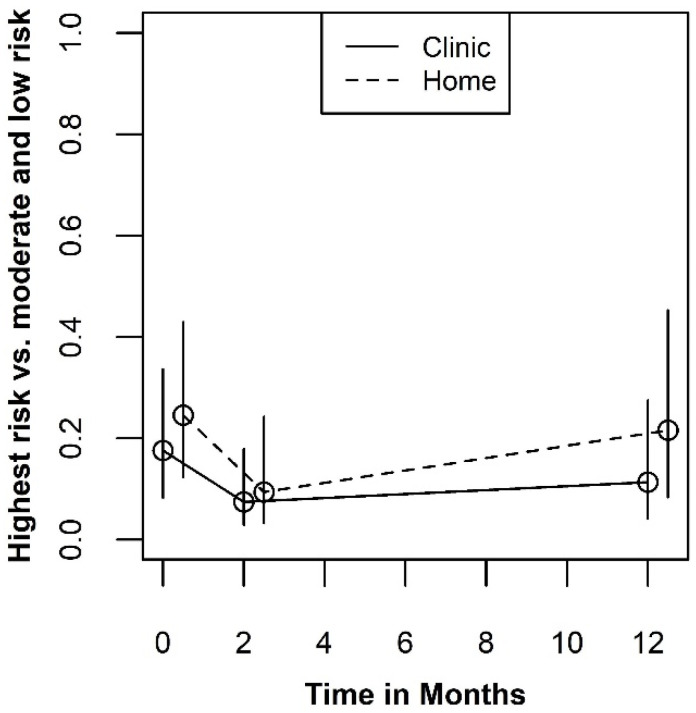
Plots for sexual behavior.

The mean plots for sexual behavior are shown in Figure 7. The plots display the fitted probabilities of being in the riskiest category verses either of the two lower risk categories. Due to the standard proportional odds assumption in cumulative logit models, the fitted probabilities for the high and medium versus low-risk categories show the same pattern. The mean plots evidence a very slight benefit for testing at home that is consistent across all three time periods. However, the Type III tests for either the main effect of testing site or the interaction with measurement occasion indicate that the effect of testing site on sexual behavior did not result in conclusive evidence against the null hypothesis (*p* = 0.389 and *p* = 0.821, respectively). Thus, these data provide very little evidence that testing site influences sexual behavior.

## 4. Discussion

We examined the effect on adolescent wellbeing and behavior of conducting HIV testing and results disclosure in a research context at home or at a health facility. Additionally, we explored the effects of the adolescent’s test result on the outcomes of interest. In our examination of the four outcomes measures, we did not find strong evidence that the decision to conduct HIV testing and disclosure of results among adolescent study participants either at home or at a clinic was very consequential. Although there were beneficial outcomes for testing at a clinic in the psychological measures, the data were far from conclusive. First, the pattern of findings was not consistent. Second, the largest benefit was seen at 12 months for the depression outcome. For the two quality of life measures, there was either very little evidence of moderation by measurement occasion or the largest effect was observed at the 2-month period, not 12-month measurement occasion. Importantly, we did not see any significant evidence for moderation of the treatment effect by baseline test result across all outcomes examined.

Researchers and program staff involved in HIV prevention work with adolescent populations may find our study results useful in developing procedures for HIV testing and disclosure of results. Consistent with Kenya’s ethical guidelines for adolescent participation in research [37,38], in our study, written informed consent and assent for research activities, including HIV testing, were obtained from parents/guardians of adolescent minors (i.e., aged 15–17) and adolescent minors, respectively. Additionally, consistent with national guidelines for HIV testing [31], adolescent minors were required to have a parent or guardian over the age of 18 in the vicinity during HIV testing and privacy and confidentiality were strictly adhered to regardless of testing venue. Additionally, on the basis of earlier work [39,40], each adolescent participant was given an opportunity to decide whether their test result would be disclosed to them while their supporting adult was present or given to their supporting adult separately. Adolescents aged 18–19 were encouraged but not required to have a supportive adult with them. Using these procedures, we successfully tested and disclosed test results to a total of 4096 adolescents. Of these, almost two-thirds were minors accompanied by a parent or guardian to whom test results pertaining to their adolescent child were also disclosed.

**Figure 8:**
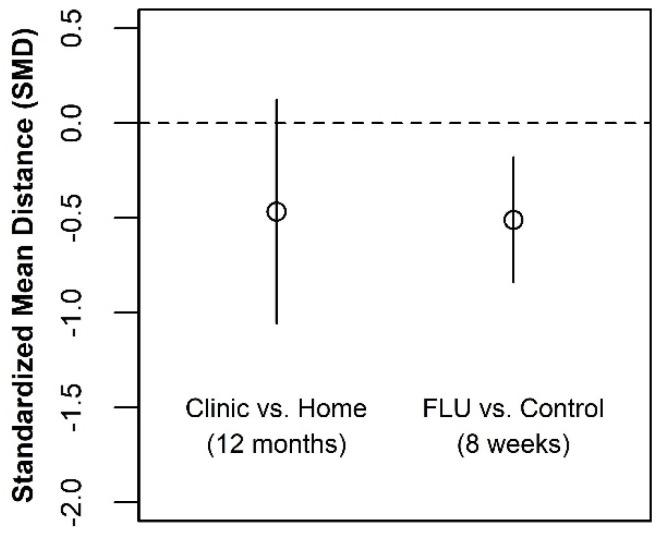
Estimated effect on depression of test venue compared with fluoxetine treatment.

To facilitate a discussion of our findings with respect to the depression measure, we have created a side-by-side comparison of our estimated effect size and a published effect size of fluoxetine (FLU), a commonly prescribed medication for depression (see Figure 8). The FLU results were obtained from a recently published meta-analysis [41]. Using the clinical trial data employed by the authors, we examined whether decisions regarding the testing site for the population of adolescents in this study could be as consequential as the decision to prescribe an anti-depressant to a patient suffering from clinical depression. The effect sizes from both studies were calculated as an SMD.

The locations of the point estimates are very similar, suggesting that our “best guess” estimate of the two effect sizes may be similar. However, the lower and upper bounds of the effect size for HIV testing at a clinic exceed the interval estimate from the meta-analysis of the drug efficacy studies. Although there is considerably more uncertainty about the consequences of HIV testing site than there is regarding the prescription of an FDA-approved anti-depressant medication, we cannot rule out the possibility that they are similar.

It is unclear why the largest benefit for the depression outcome was at the 12-month follow-up. Our data suggest that the result was driven by the HIV-positive participants who were tested at a clinic. It is possible that these participants were more likely than those who tested HIV-positive at home to become linked to treatment and support services in a timely manner and/or to still be engaged in the services at the 12-month follow-up. If so, the services may have provided psychosocial support which would result in a better depression outcome. However, these observations are speculative and more research on the topic is warranted. 

The inconsistent pattern of our results may be explained by findings from prior qualitative interviews we conducted with adolescent study participants at the 2-month follow-up, which indicated that testing venue was less important for youth than perceptions of privacy and confidentiality, being treated respectfully by service providers, and (for younger adolescents) having a parent/guardian present [29]. Other research indicates mixed findings regarding preference for facility versus home-based HIV testing among youth. For example, several studies indicate preference for and/or a higher uptake of HIV testing among youth research participants when offered at home compared with a health facility [42,43,44]. Conversely, a study among South African youth found no difference in youths’ preferences to receive an HIV test at home or in a clinic but either option was preferred compared with in a school [45]. Taken together, the implications of these mixed findings could be that for adolescent research participants, the quality of interpersonal interactions with providers during HIV testing and results disclosure may be more important than whether services are provided at home or at a clinic.

Limitations of our study include the fact that we did not examine the effects of testing and disclosure of results in a mobile clinic, at local entertainment activities often attended by youth (e.g., sports events), or via self-testing. These venues and modalities may have been preferred by some adolescents, which in turn may have affected their wellbeing at the 2-month and 12-month follow-up sessions. Another limitation is that we did not examine other characteristics of the testing and disclosure environment that have been found to be associated with HIV testing among adolescents. Examples of these characteristics include nature of family dynamics (supportive, open communication) and whether health facilities where testing was conducted had adolescent-friendly services and staff. A third limitation is that our data focuses on a community-based, rural sample and not a random or probability-based sample, which may limit generalizability of our findings to low HIV prevalence regions or areas outside of the study setting. However, a major strength of our study is that our sample included adolescents who were orphans, out of school, and/or pregnant.

## 5. Conclusions

Analogous to recent efforts to promote the inclusion of pregnant women in research [46], specific factors which can render adolescents vulnerable should be studied empirically, rather than to assume that adolescents are a vulnerable group [47]. Previous studies have reported reservations about home-based HIV testing for adolescents because of concerns about loss of privacy and confidentiality, stimulating family strife, and HIV-related stigma [48]. However, our study results suggest that the decision to conduct HIV testing and results disclosure at home or at a health facility among adolescents in HIV prevention research may not be consequential. For the promotion of responsible conduct of research with this population, this study finding will be of interest to HIV researchers who plan to include adolescents in their prevention studies, the research ethics committees who review these studies, and HIV prevention program implementers.

## Figures and Tables

**Table 1:** Baseline Characteristics of Full Sample and Follow-up Sample.

Baseline Characteristics	Full Sample		Follow-Up Sample ^a^	
	(*n* = 4096)		(*n* = 113)	
	%	*n*	%	*n*
Baseline Testing Venue				
Clinic	50.81	2081	53.98	61
Home	49.19	2015	46.02	52
Age group				
15–17 years old	61.74	2529	63.72	72
18–19 years old	38.26	1567	36.28	41
Gender				
Female	49.58	2031	51.33	58
Male	50.42	2065	48.67	55
Orphan (% yes)	41.70	1694	44.64	50
Currently in school or completed secondary (% yes)	83.25	3410	81.42	92
Ever married (% yes)	1.99	81	^b^	<5
You or partner ever pregnant (% yes)	9.45	384	^b^	<5
Attends religious services once a week or more (% yes)	53.35	2182	60.71	68
Religious affiliation				
Roman Catholic	24.95	1021	27.43	31
Protestant or other Christian	68.99	2823	64.60	73
Muslim, no religion, or other	6.06	248	7.96	9
Subcounty location				
Subcounty 1	26.93	1103	28.32	32
Subcounty 2	35.47	1453	28.32	32
Subcounty 3	37.60	1540	43.36	49

^a^ Selected to participate in the follow-up. ^b^ Percentage is too small to report.

## Data Availability

Consistent with the consent form used and advice of our IRB, data will not be made available.

## References

[1] Jaspan H.B., Cunningham C.K., Tucker T.J., Wright P.F., Self S.G., Sheets R.L., Rogers A.S., Bekker L.G., Wilson C.M., Duerr A. (2008). Inclusion of adolescents in preventive HIV vaccine trials: Public health policy and research design at a crossroads. J. Acquir. Immune Defic. Syndr..

[2] Nagata J.M., Ferguson B.J., Ross D.A. (2018). Minding the Gap: Setting Research Priorities Related to HIV Testing, Treatment, and Service Delivery Among Adolescents. J. Adolesc. Health.

[3] Pomfret S., Karim Q.A., Benatar S.R. (2010). Inclusion of adolescent women in microbicide trials: A public health imperative!. Public Health Ethics.

[4] Kapogiannis B.G., Handelsman E., Ruiz M.S., Lee S. (2010). Introduction: Paving the way for biomedical HIV prevention interventions in youth. J. Acquir. Immune Defic. Syndr..

[5] Armstrong A., Nagata J.M., Vicari M., Irvine C., Cluver L., Sohn A.H., Ferguson J., Caswell G., Njenga L.W., Oliveras C. (2018). A Global Research Agenda for Adolescents Living With HIV. J. Acquir. Immune Defic. Syndr..

[6] Idele P., Gillespie A., Porth T., Suzuki C., Mahy M., Kasedde S., Luo C. (2014). Epidemiology of HIV and AIDS among adolescents: Current status, inequities, and data gaps. J. Acquir. Immune Defic. Syndr..

[7] UNAIDS Data 2020—AIDSinfo Data Sheet, People living with HIV—All Adolescents (10–19), Regional Data Sheet. http://aidsinfo.unaids.org/.

[8] Singh J.A., Siddiqi M., Parameshwar P., Chandra-Mouli V. (2019). World Health Organization Guidance on ethical considerations in planning and reviewing research studies on sexual and reproductive health in adolescents. J. Adolesc. Health.

[9] MacQueen K.M., Karim Q.A. (2007). Practice brief: Adolescents and HIV clinical trials: Ethics, culture, and context. J. Assoc. Nurses AIDS Care.

[10] Singh J.A., Karim S.S.A., Karim Q.A., Mlisana K., Williamson C., Gray C., Govender M., Gray A. (2006). Enrolling adolescents in research on HIV and other sensitive issues: Lessons from South Africa. PLoS Med..

[11] Slack C., Strode A., Fleischer T., Gray G., Ranchod C. (2007). Enrolling adolescents in HIV vaccine trials: Reflections on legal complexities from South Africa. BMC Med. Ethics.

[12] Bekker L.-G., Slack C., Lee S., Shah S., Kapogiannis B. (2014). Ethical issues in adolescent HIV research in resource-limited countries. J. Acquir. Immune Defic. Syndr..

[13] Knopf A.S., Gilbert A.L., Zimet G.D., Kapogiannis B.G., Hosek S.G., Fortenberry J.D., Ott M.A. (2017). Moral conflict and competing duties in the initiation of a biomedical HIV prevention trial with minor adolescents. AJOB Empir. Bioeth..

[14] Nelson R.M., Lewis L.L., Struble K., Wood S.F. (2010). Ethical and regulatory considerations for the inclusion of adolescents in HIV biomedical prevention research. J. Acquir. Immune Defic. Syndr..

[15] Slack C., Strode A., Grant C., Milford C. (2005). Implications of the ethical-legal framework for adolescent HIV vaccine trials—Report of a consultative forum. S. Afr. Med. J..

[16] Day S., Kapogiannis B.G., Shah S.K., Wilson E.C., Ruel T.D., Conserve D.F., Strode A., Donenberg G.R., Kohler P., Slack C. (2020). Adolescent participation in HIV research: Consortium experience in low and middle-income countries and scoping review. Lancet HIV.

[17] Luseno W.K., Hallfors D.D., Cho H., Iritani B.J., Adze J., Rusakaniko S., Mbai I., Milimo B., Hobbs M. (2014). Use of HIV and HSV-2 biomarkers in sub-Saharan adolescent prevention research: A comparison of two approaches. J. Prim. Prev..

[18] Birdthistle I.J., Floyd S., Machingura A., Mudziwapasi N., Gregson S., Glynn J.R. (2008). From affected to infected? Orphanhood and HIV risk among female adolescents in urban Zimbabwe. AIDS.

[19] Cowan F., Pettifor A. (2009). HIV in adolescents in sub-Saharan Africa. Curr. Opin. HIV AIDS.

[20] Cowan F.M., Pascoe S.J., Langhaug L.F., Dirawo J., Chidiya S., Jaffar S., Mbizvo M., Stephenson J.M., Johnson A.M., Power R.M. (2008). The Regai Dzive Shiri Project: A cluster randomised controlled trial to determine the effectiveness of a multi-component community-based HIV prevention intervention for rural youth in Zimbabwe—study design and baseline results. Trop. Med. Int. Health.

[21] Bandason T., Dauya E., Dakshina S., McHugh G., Chonzi P., Munyati S., Weiss H.A., Simms V., Kranzer K., Ferrand R.A. (2018). Screening tool to identify adolescents living with HIV in a community setting in Zimbabwe: A validation study. PLoS ONE.

[22] Bekolo C.E., Yimdjo Fogue T.D., Williams T.D.A. (2018). Feasibility of integrating HIV testing into local youth development p rogrammes in Cameroon. Pan Afr. Med. J..

[23] Kadede K., Ruel T., Kabami J., Ssemmondo E., Sang N., Kwarisiima D., Bukusi E., Cohen C.R., Liegler T., Clark T.D. (2016). Increased adolescent HIV testing with a hybrid mobile strategy in Uganda and Kenya. AIDS.

[24] Shanaube K., Schaap A., Chaila M.J., Floyd S., Mackworth-Young C., Hoddinott G., Hayes R., Fidler S., Ayles H. (2017). Community intervention improves knowledge of HIV status of adolescents in Zambia: Findings from HPTN 071-PopART for youth study. AIDS.

[25] Ssebunya R.N., Wanyenze R.K., Namale L., Lukolyo H., Kisitu G.P., Nahirya-Ntege P., Kekitiinwa A. (2018). Prevalence and correlates of HIV testing among adolescents 10–19 years in a post-conflict pastoralist community of Karamoja region, Uganda. BMC Public Health.

[26] Sennott C., Yeatman S. (2016). Surprising results: HIV testing and changes in contraceptive practices among young women in Malawi. J. Biosoc. Sci..

[27] Baird S., Gong E., McIntosh C., Özler B. (2014). The heterogeneous effects of HIV testing. J. Health Econ..

[28] Luseno W.K., Field S.H., Iritani B.J., Odongo F.S., Kwaro D., Amek N.O., Rennie S. (2021). Pathways to Depression and Poor Quality of Life Among Adolescents in Western Kenya: Role of Anticipated HIV Stigma, HIV Risk Perception, and Sexual Behaviors. AIDS Behav..

[29] Simons-Rudolph A.P., Iritani B.J., Odongo F.S., Rennie S., Gilbertson A., Kwaro D., Luseno W.K. (2020). Adolescent perceptions about participating in HIV-related research studies. Child. Youth Serv. Rev..

[30] UNAIDS Data 2019—AIDSinfo Data Sheet, HIV Prevalence—Young People (15–24), Kenya Sub-National Data Sheet. http://aidsinfo.unaids.org/.

[31] National AIDS and STI Control Programme (NASCOP) Ministry of Health Kenya (2015). Guidelines for HIV Testing Services in Kenya.

[32] Van Dam N.T., Earleywine M. (2011). Validation of the Center for Epidemiologic Studies Depression Scale--Revised (CESD-R): Pragmatic depression assessment in the general population. Psychiatry Res..

[33] Skevington S.M., Lotfy M., O’Connell K.A. (2004). The World Health Organization’s WHOQOL-BREF quality of life assessment: Psychometric properties and results of the international field trial A Report from the WHOQOL Group. Qual. Life Res..

[34] World Health Organization (WHO) (2004). The World Health Organization Quality of Life (WHOQOL)-BREF.

[35] Allison P.D. (2001). Missing Data.

[36] Molenberghs G., Verbeke G. (2006). Models for Discrete Longitudinal Data.

[37] National AIDS and STI Control Programme (NASCOP) & Kenya Medical Research Institute (KEMRI) (2015). Guidelines for Conducting Adolescents Sexual and Reproductive Health Research in Kenya.

[38] Republic of Kenya (2005). Guidelines for Ethical Conduct of Biomedical Research Involving Human Subjects in Kenya.

[39] Groves A.K., Hallfors D.D., Iritani B.J., Rennie S., Odongo F.S., Kwaro D., Amek N., Luseno W.K. (2018). “I think the parent should be there because no one was born alone”: Kenyan adolescents’ perspectives on parental involvement in HIV research. Afr. J. AIDS Res..

[40] Luseno W.K., Iritani B.J., Maman S., Mbai I., Ongili B., Otieno F.A., Hallfors D.D. (2019). “If the mother does not know, there is no way she can tell the adolescent to go for drugs”: Challenges in promoting health and preventing transmission among pregnant and parenting Kenyan adolescents living with HIV. Child. Youth Serv. Rev..

[41] Zhou X., Teng T., Zhang Y., Del Giovane C., Furukawa T.A., Weisz J.R., Li X., Cuijpers P., Coghill D., Xiang Y. (2020). Comparative efficacy and acceptability of antidepressants, psychotherapies, and their combination for acute treatment of children and adolescents with depressive disorder: A systematic review and network meta-analysis. Lancet Psychiatry.

[42] Inwani I., Chhun N., Agot K., Cleland C.M., Rao S.O., Nduati R., Kinuthia J., Kurth A.E. (2020). Preferred HIV Testing Modalities Among Adolescent Girls and Young Women in Kenya. J. Adolesc. Health.

[43] Sharma M., Ying R., Tarr G., Barnabas R. (2015). Systematic review and meta-analysis of community and facility-based HIV testing to address linkage to care gaps in sub-Saharan Africa. Nature.

[44] Doherty T., Tabana H., Jackson D., Naik R., Zembe W., Lombard C., Swanevelder S., Fox M.P., Thorson A., Ekström A.M. (2013). Effect of home based HIV counselling and testing intervention in rural South Africa: Cluster randomised trial. BMJ.

[45] Chetty-Makkan C.M., Hoffmann C.J., Charalambous S., Botha C., Ntshuntshe S., Nkosi N., Kim H.-Y. (2021). Youth Preferences for HIV Testing in South Africa: Findings from the Youth Action for Health (YA4H) Study Using a Discrete Choice Experiment. AIDS Behav..

[46] Krubiner C.B., Faden R.R., Cadigan R.J., Gilbert S.Z., Henry L.M., Little M.O., Mastroianni A.C., Namey E.E., Sullivan K.A., Lyerly A.D. (2016). Advancing HIV research with pregnant women: Navigating challenges and opportunities. AIDS.

[47] Luna F. (2019). Identifying and evaluating layers of vulnerability—A way forward. Dev. World Bioeth..

[48] Perriat D., Plazy M., Gumede D., Boyer S., Pillay D., Dabis F., Seeley J., Orne-Gliemann J. (2018). “If you are here at the clinic, you do not know how many people need help in the community”: Perspectives of home-based HIV services from health care workers in rural KwaZulu-Natal, South Africa in the era of universal test-and-treat. PLoS ONE.

